# Engineering of SnO_2_–Graphene Oxide Nanoheterojunctions for Selective Room-Temperature Chemical Sensing and Optoelectronic Devices

**DOI:** 10.1021/acsami.0c09178

**Published:** 2020-07-22

**Authors:** Eleonora Pargoletti, Umme H. Hossain, Iolanda Di Bernardo, Hongjun Chen, Thanh Tran-Phu, Gian Luca Chiarello, Josh Lipton-Duffin, Valentina Pifferi, Antonio Tricoli, Giuseppe Cappelletti

**Affiliations:** † Dipartimento di Chimica, Università degli Studi di Milano, via Golgi 19, Milano 20133, Italy; ‡ Consorzio Interuniversitario Nazionale per la Scienza e Tecnologia dei Materiali (INSTM), Via Giusti 9, Firenze 50121, Italy; § Department of Electronic Materials Engineering, Research School of Physics and Engineering, The Australian National University, Canberra Australian Capital Territory 2601, Australia; ∥ Nanotechnology Research Laboratory, College of Engineering and Computer Science, The Australian National University, Canberra Australian Capital Territory 2601, Australia; ⊥ Institute for Future Environments (IFE), Central Analytical Research Facility (CARF), Queensland University of Technology(QUT), Brisbane 4000, Australia

**Keywords:** nanoheterojunctions, graphene oxide, tin dioxide, UV photodetectors, room-temperature chemoresistive sensing, selectivity

## Abstract

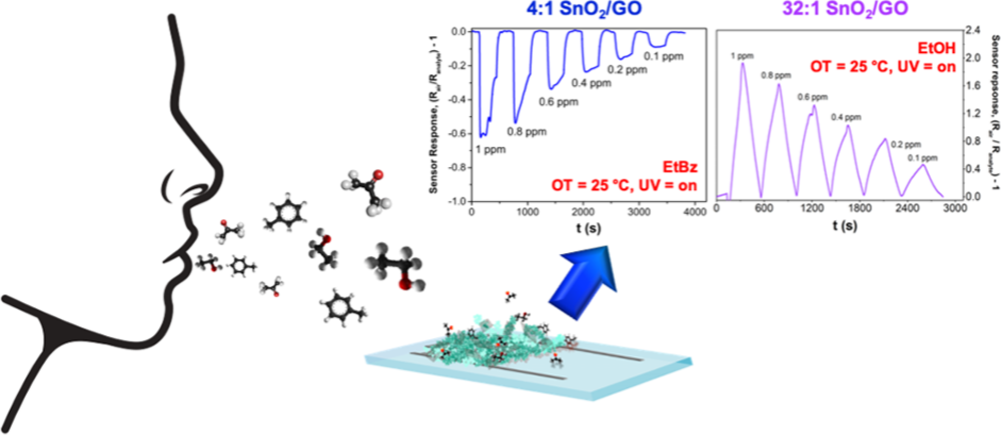

The development of high-performing sensing materials, able to detect ppb-trace concentrations of volatile organic compounds (VOCs) at low temperatures, is required for the development of next-generation miniaturized wireless sensors. Here, we present the engineering of selective room-temperature (RT) chemical sensors, comprising highly porous tin dioxide (SnO_2_)–graphene oxide (GO) nanoheterojunction layouts. The optoelectronic and chemical properties of these highly porous (>90%) p–n heterojunctions were systematically investigated in terms of composition and morphologies. Optimized SnO_2_–GO layouts demonstrate significant potential as both visible–blind photodetectors and selective RT chemical sensors. Notably, a low GO content results in an excellent UV light responsivity (400 A W^–1^), with short rise and decay times, and RT high chemical sensitivity with selective detection of VOCs such as ethanol down to 100 ppb. In contrast, a high concentration of GO drastically decreases the RT response to ethanol and results in good selectivity to ethylbenzene. The feasibility of tuning the chemical selectivity of sensor response by engineering the relative amount of GO and SnO_2_ is a promising feature that may guide the future development of miniaturized solid-state gas sensors. Furthermore, the excellent optoelectronic properties of these SnO_2_–GO nanoheterojunctions may find applications in various other areas such as optoelectronic devices and (photo)electrocatalysis.

## Introduction

The development of ultraminiaturized and low-power consumption sensors for monitoring of volatile organic compound (VOC) concentrations is becoming increasingly important because of the rapid pace of emission of potentially toxic VOCs in urban areas and their role as biomarkers in noninvasive medical diagnostics.^1^ Many VOCs are highly toxic with potential carcinogenic, mutagenic, and teratogenic functions at low concentrations. They can also contribute to atmospheric pollution, such as photochemical smog and destruction of the ozone layer.^2^ Recently, significant attention has been devoted to the BTEX compounds, namely, benzene, toluene, ethylbenzene, and xylene, because of their increasing release in various industrial processes.^2−4^ Quite a few VOCs are also present in the human breath and correlated with several metabolic processes. Specifically, the monitoring of VOCs spontaneously released by the body is increasingly considered as a promising path for noninvasive medical diagnostics and health monitoring.^5,6^ For instance, abnormal concentrations of acetone (>1800 ppb) can be related to type 1 diabetes, where the standard concentrations in people not affected by this illness are 300–900 ppb.^7^ Similarly, a marked presence of ethanol and acetone are related to nonalcoholic fatty liver disease and hepatic steatosis.^8^ Ethylbenzene as well, apart from being a BTEX compound, has been recently recognized as one of the potential biomarkers for lung cancer detection (0.04 ppb in healthy humans *vs* 0.11 ppb in ill patients).^9−11^ Hence, the need for frequent VOC monitoring with deployable, portable, or wearable detectors has attracted a widespread interest in the development of few millimetres in size wireless sensor devices.^5^

Chemoresistive gas sensors, based on nanostructured metal oxide semiconductors (MOS), are a promising technology for low-concentration detection of VOCs with superior miniaturization potential to established analytical techniques such as proton-transfer reaction mass spectrometry and gas chromatography.^12^ Development of MOS sensors is held back from few fundamental challenges related to the sensing material, including high operating temperatures (OT) (200–400 °C)^13−15^ and their difficulty in achieving selectivity in multiple gas environments.^1^ Much effort is focused to address the above challenges. Various recent studies report the design and fabrication of MOS (*e.g.*, ZnO,^16−18^ NiO,^13^ and WO_3_^19^) with unique nanoarchitectures that enable low-temperature sensing.^13−15^ However, the lower limit of detection is often at the ppm level and thus very high for many applications, including detection of important biomarkers for breath analysis.^14,15^

The use of heterojunctions between metal oxides^13^ or by coupling them with carbonaceous materials^20,21^ has been reported as a path to improve the gas sensing performance of MOS, with particular merits for room-temperature (RT) detection under light irradiation. Notably, graphene materials possess several promising features such as thermoelectric conductivity and mechanical strength,^9^ which can enhance the sensing behavior of MOS by formation of nanoscale heterojunctions. Reduced graphene oxide (rGO) has been widely investigated for electrochemical applications and offers some potential for gas sensing.^22−24^ For instance, Meng et al.^25^ recently described a ternary sensing materials made of ZnO–rGO sensitized with graphitic carbon nitride that exhibits superior ethanol vapor sensing, reaching a 9-fold higher response than pure ZnO. Similarly, a newly ternary nanocomposite material comprising Au, SnO_2_, and rGO has been reported to successfully detect low ppm concentrations of ethanol at OT down to 50 °C. This was attributed to the synergistic effects arising between SnO_2_ and rGO that were further enhanced by decoration with gold nanoparticles.^26^ Ultimately, Yuan et al.^27^ synthesized a sandwich-like composite consisting of a double layer of Co_3_O_4_ and rGO, reporting a 5-fold increase in response to 100 ppm of triethylamine with respect to a conventional bare Co_3_O_4_ semiconductor. In contrast, pure graphene oxide (GO) has been scarcely reported for this application^24,28,29^ because of its less defective structure and surface chemistry.^28,30^ Nevertheless, its oxygen-rich functional groups can be the anchor points that help the further growth of MOS nanoparticles. Particularly, if the adopted metal oxide behaves as an n-type semiconductor, the conceivable formation of p(GO)^31,32^–n(MOS) heterojunction may be hypothesized.

Here, we report the fabrication of an ultraporous nanoheterojunction network of SnO_2_ and GO, demonstrating the effective engineering of their chemical sensing and photoresponsive properties tuning the p- and n-type nanodomain fraction. The photo- and chemical sensing features of these nanoheterojunctions were systematically investigated, achieving new insights into the role played by the GO in the enhancement of the RT sensor response and the selectivity toward a particular VOC. Specifically, three different volatile compounds, i.e. ethanol (EtOH), acetone and the less-studied ethylbenzene (EtBz), were adopted as target molecules. Notably, we observed that a small amount of GO leads to the formation of electron-depleted nanoheterojunctions with superior electron–hole separation efficiency. These nanocomposites are able to selectively detect ethanol concentrations down to 100 ppb at RT. Conversely, the increase of GO content hinders ethanol sensing and favors ethylbenzene detection, providing for the first time a mechanism to tailor MOS sensor selectivity. We demonstrated that this optimal nanocomposite structure provides excellent photo- and chemical responses, showcasing their applicability as both visible–blind UV photodetectors and selective RT VOC solid-state sensors.

## Experimental Section

All the chemicals were of reagent-grade purity; Milli-Q water was utilized. All the adopted reagents were purchased from Sigma-Aldrich.

### Synthesis of Pristine Oxides and Hybrid SnO_2_–GO Compounds

GO was prepared through modified Hummers method already reported in the literature.^29,33^ For the composite materials, SnO_2_–GO, the adopted synthetic route was the same described in our previous works^28,29^ with different starting salt precursor-to-GO weight ratios (*i.e.*, 4:1 and 32:1 SnO_2_–GO since with the other intermediate ratios, lower sensing performances were obtained, as deeply discussed in our previous study).^29^ For the sake of comparison, pure SnO_2_ was prepared through the same synthetic route, without the addition of GO.

### Powder Physicochemical Characterizations

X-ray diffraction (XRD) analyses were performed on a Philips PW 3710 Bragg–Brentano goniometer as described elsewhere,^29^ collecting spectra between 10 and 80° with a step size of 0.1°.

Raman spectra were collected on a Renishaw inVia micro-Raman spectrometer, as reported in our previous study.^29^

The Brunauer–Emmett–Teller (BET)-specific surface area was determined by a multipoint BET method. Desorption isotherms were used to determine the total pore volume using the Barrett–Joyner–Halenda (BJH) method, as stated elsewhere.^29^

The morphology was investigated by using a Zeiss Ultraplus (field-emission scanning electron microscopy, FESEM) at 3 kV coupled with an energy-dispersive X-ray (EDX) spectrophotometer for elemental analysis. Transmission electron microscopy (TEM) analyses were carried out on Hitachi H7100FA at 100 kV. The TEM grids were prepared as already described.^29^

Thermogravimetric analyses were carried out by means of a Mettler Toledo Star and System TGA/DSC 3+ under air atmosphere (5 °C min^–1^ from 30 to 800 °C).

X-ray photoemission spectroscopy (XPS) data were collected in a Thermo Fisher Kratos Axis Supra photoelectron spectrometer at the Central Analytical Research Facility of the Queensland University of Technology (Brisbane, Australia). The apparatus is provided by a monochromated Al kα source (1486.7 eV), and the spectra were calibrated with respect to their Fermi level. Survey spectra were acquired at pass energy 160 and high-resolution spectra at pass energy 20.

Powder optical band gaps were evaluated by Kubelka–Munk elaboration. Diffuse reflectance spectroscopy (DRS) spectra were measured on a UV/vis spectrophotometer Shimadzu UV-2600 equipped with an integrating sphere; a “total white” BaSO_4_ was used as a reference. The porosity of SnO_2_ nanoparticle networks of the films was estimated from the optical density and SEM visible thickness as suggested by Bo et al.^34^ adopting an absorption coefficient of 3.08 × 10^7^ m^–1^ (at 312 nm for all the powders).

Electrochemical impedance spectroscopy (EIS) experiments were carried out as reported in our previous study.^29^

### Deposition on Pt-Interdigitated Electrodes

Powders were deposited on glass substrates topped with Pt interdigitated electrodes (Pt-IDEs) by a simple hot-spray method reported elsewhere.^28^ Therefore, the tested IDEs were prepared by adopting pristine SnO_2_, hybrid 4:1, and 32:1 SnO_2_–GO powders.

### Photodetector Measurements and Gas Sensing Tests

For photodetector tests, photo- and dark-currents were measured at 25 °C with an LCS-100 Series Small Area Solar Simulator (Newport Co.). The electrode active surface was equal to 0.4 cm^–2^, and the irradiation power at 312 nm was 1.5 μW cm^–2^. The responsivity and detectivity were, then, calculated according to the equations reported elsewhere.^34^ Regarding NO_2_, ethanol (EtOH), acetone and ethylbenzene (EtBz) sensing, O_2_ (BOC Ltd), and N_2_ (BOC Ltd) were controlled by a mass flow controller (Bronkhorst), with a total gas flow rate of 0.5 L min^–1^. The target gas (10 ppm in N_2_, Coregas) was diluted to 1 ppm and lower concentrations by using the simulated air (0.1 L min^–1^ O_2_ + 0.4 L min^–1^ N_2_, BOC Ltd) before purging into the chamber, keeping the total flow rate constant. The temperature of the hot plate in the gas sensing chamber (Linkam) was controlled by a temperature controller and, when the OT was lowered (equal or below 150 °C), UV light was also exploited. The samples were illuminated through a quartz window by a solar simulator (NewSpec, LCS-100) with an FGUV5-UV–Ø25 mm UG5 Colored Glass Filter (AR Coated: 290–370 nm, Thorlabs Inc). For the gas sensing tests, the adopted experimental procedure is finely described in previous works of some authors of the present paper.^28,29^ Furthermore, tests conducted under controlled relative humidity (RH of *ca*. 80%) were carried out by exploiting a bubbler through which the target gas evaporated.

To shed light on the intrinsic materials electrical features, Figure S1 displays both the resistance variations upon purging a representative VOC as ethanol for pure and hybrid compounds, adopting the same operative conditions used during sensing measurements (temperature, UV, and applied bias). Besides, Figure S1c shows the resistance of pure GO.

## Results and Discussion

### Synthesis of a SnO_2_–GO Nanoheterojunction Network

The graphite conversion into the GO material and the subsequent formation of nanoheterojunctions with a three-dimensional SnO_2_ network have been verified by a combination of physical and chemical characterizations. Two different relative GO concentrations of SnO_2_–GO 4:1 and 32:1 were investigated to evaluate the potential optoelectronic and chemoresistive performances of the SnO_2_–GO nanoheterojunctions. These SnO_2_–GO ratios were selected with respect to the previous study on ZnO–GO nanoscale heterojunctions.^29^

**Figure 1:**
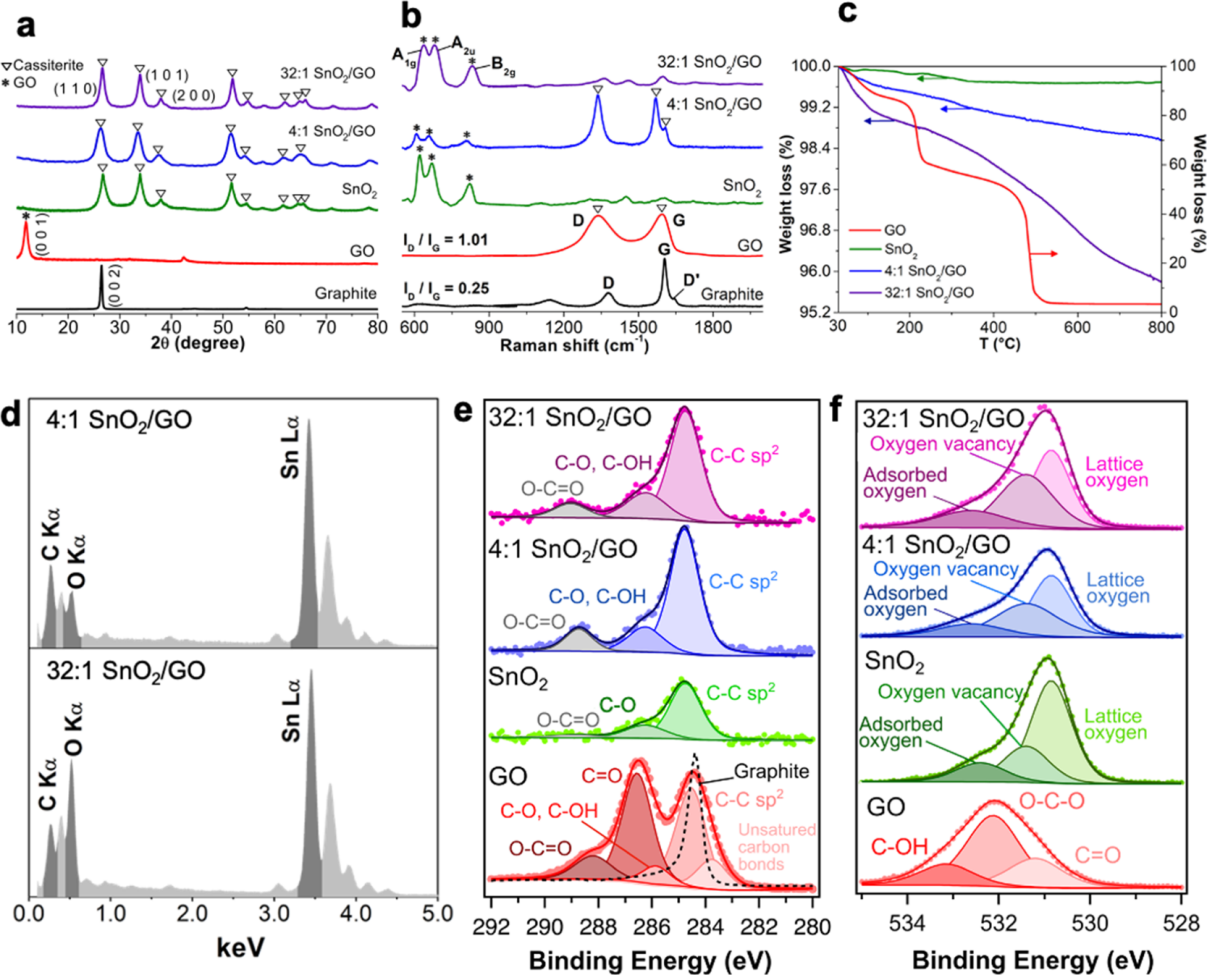
(a) XRD patterns of graphite, GO, pure SnO_2_, and hybrid SnO_2_–GO samples. (b) Raman spectra of all the investigated samples. (c) TGA spectra of GO, pure, and hybrid nanopowders. (d) EDX spectra of 4:1 SnO_2_–GO and 32:1 SnO_2_–GO. XP spectra of (e) C 1s and (f) O 1s regions of graphite, GO, 4:1, and 32:1 SnO_2_–GO.

**Figure 2:**
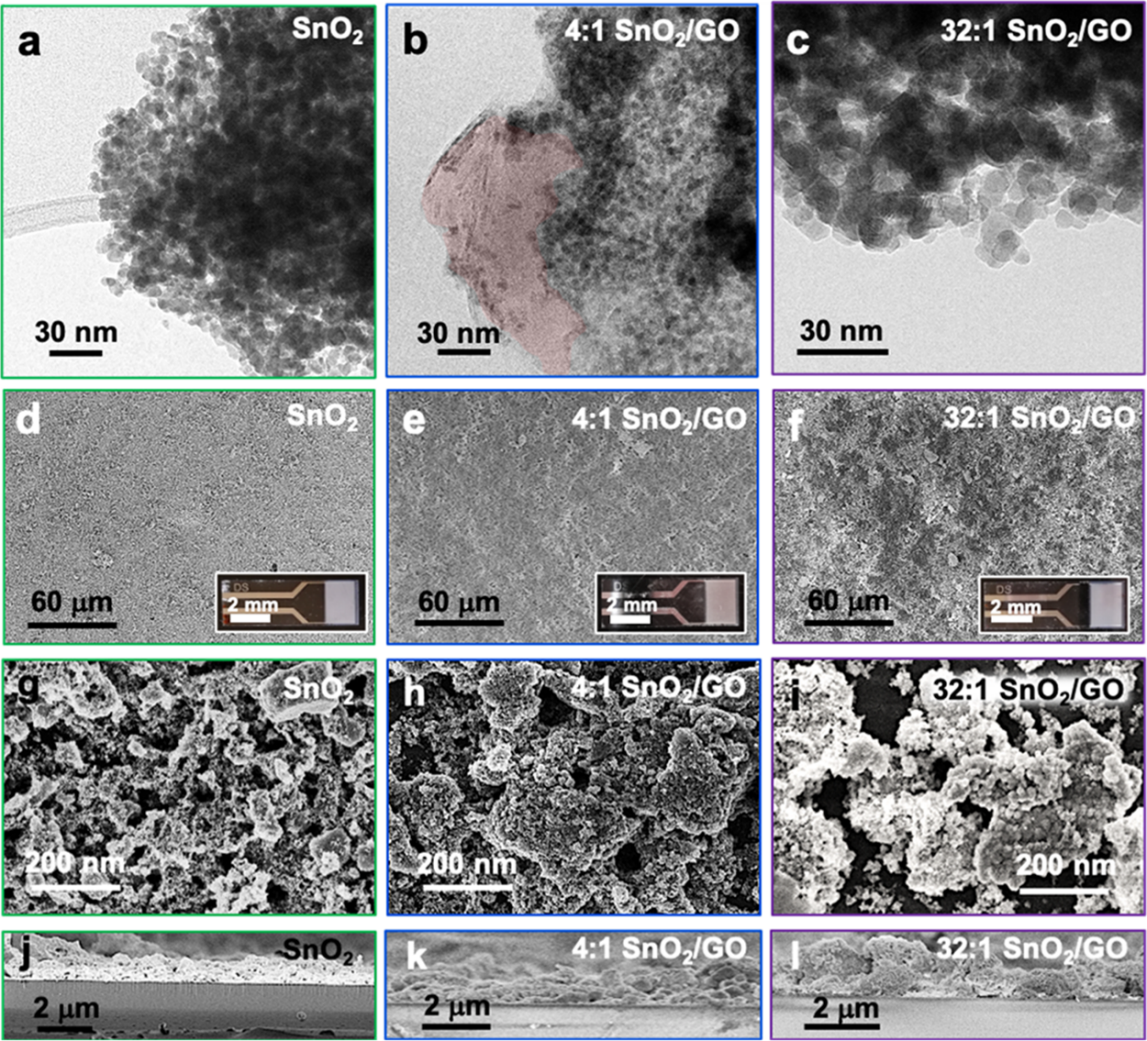
(a–c) TEM images of pristine SnO_2_ and hybrid SnO_2_–GO compounds. (b) Presence of GO was highlighted in red. (d–i) Top-view FESEM micrographs and (j–l) cross-sectional images of both pure and composite samples. Insets: photos of the relative IDEs.

Figure 1a,b shows a comparison of the XRD patterns and Raman spectra of the pristine graphite and GO, along with the structural data of bare SnO_2_ and the synthesized SnO_2_–GO composites. The effective transformation of graphite material into GO was assessed by both the main GO diffraction peak (at 2θ value of 12°) and the intensity increase of the ratio between the D and G Raman bands up to 1.01 of GO *versus* 0.25 of the precursor graphite (Figure 1a,b, red spectra).^28,29^ Indeed, during the oxidation process, oxygen functional groups are introduced into the graphitic chain, causing either an increase of the D band intensity^28,35^ or a small shift (*ca*. 25 cm^–1^ upward for G band and 45 cm^–1^ downward for D band) of the band positions,^28^ because of the achievement of a highly defective structure.^36,37^ Moreover, the gradual integration of GO nanodomains into the SnO_2_ matrix was revealed by both the presence of Raman bands relative to the GO material (Figure 2b, blue and violet spectra) and the very small XRD crystallite diameter (*ca*. 5–8 nm; Figure 1a and Table 1, fourth column). Indeed, the crystallite size of the SnO_2_–GO nanoheterojunctions resembles much more the GO one (11 nm, Table 1), underlining the effective integration of the carbonaceous material into the metal oxide network.

**Table 1:** Surface Area (*S*_BET_), Total Pore Volume (*V*_tot. pores_), Crystallite Domain Size by XRD Analysis (⟨*d*^XRD^⟩), Optical Band Gap (*E*_g_, by Kubelka–Munk Extrapolation), Film Thickness (by Cross-Sectional SEM), and Film Porosity Percentage (Obtained by Means of UV/vis Spectroscopy Technique)

sample	*S*_BET_ (m^2^ g^–1^)	*V*_tot. pores_ (cm^3^ g^–1^)	⟨*d*^XRD^⟩ (nm)	*E*_g_ (eV)	film thickness (μm)	% film porosity
graphite	11	0.030	27	-	-	-
GO	30	0.020	11	-	-	-
SnO_2_	67	0.210	15	3.6	1.8 ± 0.2	93 ± 1
4:1 SnO_2_–GO	29	0.070	5	3.0	1.2 ± 0.4	97 ± 1
32:1 SnO_2_–GO	55	0.133	8	3.4	1.4 ± 0.4	94 ± 2

Thermogravimetric analysis reveals that the hybrid samples are very stable (Figure 1c) with a mass loss of only ∼4% up to temperatures of 800 °C, resembling the typical behavior of the pristine SnO_2_. This indicates that the presence of metal oxide prevents the decomposition of the underneath GO. On the contrary, pure GO (Figure 1c, red line) decomposes in several stages, ascribable to different processes, such as (i) the loss of moisture and interstitial water between 60 and 110 °C; (ii) the pyrolysis of labile oxygen-containing groups with the generation of CO, CO_2_, and water^38^ at 200 °C; and (iii) the breakage of sp^2^ carbon bonds at around 480 °C.^29,37^ Furthermore, the presence of tin in the hybrid samples was confirmed by EDX data (Figure 1d). The surface composition of the SnO_2_–GO materials was further investigated by XPS and BET–BJH analyses. The C 1s and O 1s core-level high-resolution spectra of the GO compound (Figure 1e,f, red spectra) were discussed recently.^28,29,39^ Besides, the C 1s region of both pure and SnO_2_–GO compounds shows three components, referable to C–C sp^2^ (284.75 eV), C–O/C–OH (286.20 eV), and O–C=O (289.00 eV) bonds.^40,41^ While the last two carbon peaks are mostly attributed to adventitious CO_2_, its enhanced presence in the nanoheterojunctions is indicative of the presence of GO.^28,29^Figure 1f shows the O 1s core-level high-resolution spectra, which can be deconvoluted into three components centered at around 530.75, 531.40, and 532.60 eV. These bands correspond respectively to (i) lattice oxygen anions (O^2–^) in the cassiterite lattice, (ii) oxygen ions (O^2–^ and O^–^) in the oxygen-deficient regions, caused by oxygen vacancies, and (iii) adsorbed oxygen species (especially water molecules).^42,43^ The relative amount of oxygen vacancies in the SnO_2_–GO compounds is higher than in the pure SnO_2_, suggesting a more defective structure as a result of the GO integration into the metal oxide matrix. Furthermore, the specific surface areas (Table 1, second column) of the nanoheterojunctions increases with decreasing GO content (29 and 55 m^2^ g^–1^ for 4:1 and 32:1 SnO_2_–GO, respectively), approaching that of the pure SnO_2_ (67 m^2^ g^–1^).^28^ The same trend was observed for the total pore volume data (Table 1, third column), where 4:1 SnO_2_–GO has a value (0.070 cm^3^ g^–1^) comparable to that of pure GO (0.020 cm^3^ g^–1^), whereas 32:1 SnO_2_–GO exhibits a larger pore volume and size distribution (Figure S2b and inset of Figure S2a), because of the increasing amount of tin dioxide. Figure S2b shows a rise in pore numbers with diameter above 20 nm. Besides, by evaluating the hysteresis loop of the BET desorption isotherms (Figure S2a), we can observe the presence of slit-shaped pores for the GO and the hybrid materials, while bare SnO_2_ possesses bottleneck pores, in line with previous studies on SnO_2_–GO.^28^

Figure 2 shows the morphology of the pristine and composite samples by TEM and FESEM. Notably, both the 4:1 and 32:1 SnO_2_–GO ratios seem to be composed of spherical nanoparticles with dimensions of around 8–10 nm (Figure 2b,c), which are larger than the pure oxide ones, having a size of ∼4–6 nm (Figure 2a).^28^ Interestingly, with 4:1 lowest ratio, the presence of underneath GO is still clearly observable (Figure 2b). Also scanning electron micrographs display the presence of spherical agglomerates with dimensions of hundreds of nanometers for all the three SnO_2_-based samples (Figure 2g–i).

Overall, this set of characterizations indicates that the gradual coverage of the GO sheets by SnO_2_ is achieved, creating strong bonds between the graphene and the metal oxide nanoparticles. This tunable coverage can influence the structural and surface properties, the morphology, and crystal size of the as-prepared powders, thus affecting their behavior as photo- and chemical sensing materials.

### Optoelectronic and Chemical Sensing Properties

**Figure 3:**
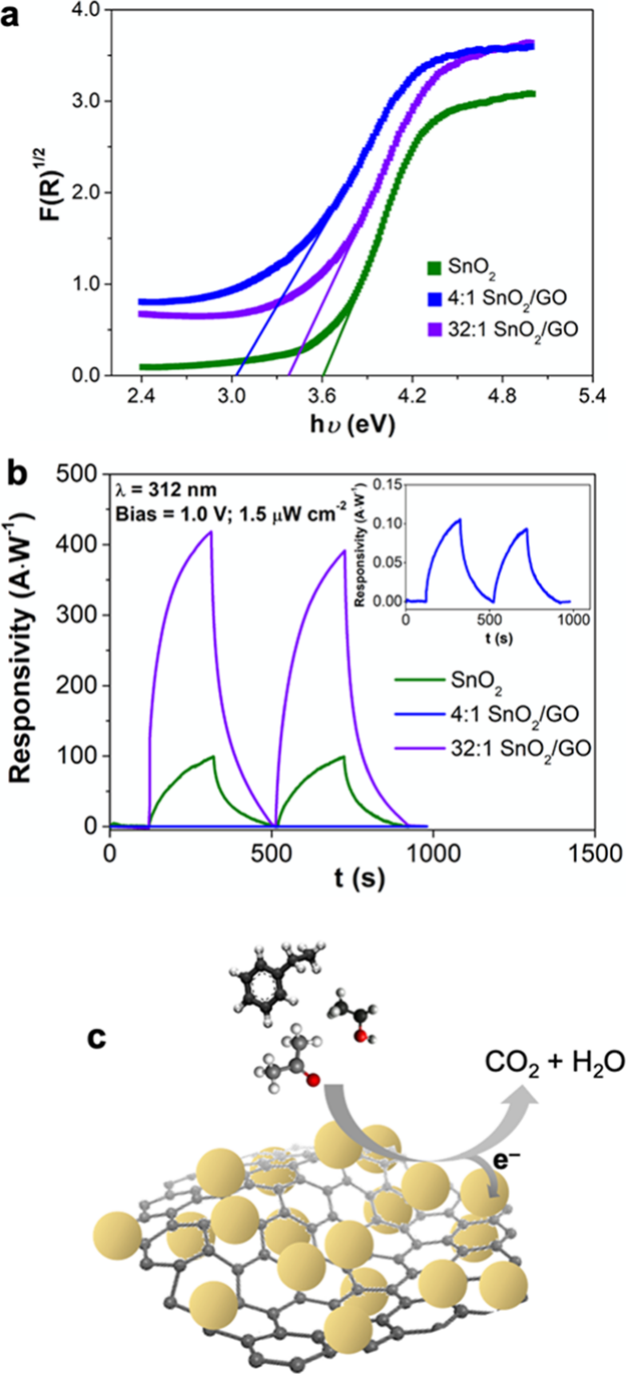
(a) Band gap values determined by Kubelka–Munk elaboration. (b) Dynamics of photodetector responsivity for all the Sn-based compounds (λ = 312 nm, light power density = 1.5 μW cm^–2^, and applied bias = +1.0 V). (c) Schematic illustration of VOC sensing by hybrid SnO_2_–GO nanomaterials.

The formation of nanoscale heterojunctions is a promising approach to improve chemical sensing at low temperatures by photoexcitation and separation of reactive electron/hole couples.^13,28,44−46^ The optical properties of the tin dioxide-containing compounds were initially investigated by DRS. Figure 3a shows the Kubelka–Munk conversion of the DRS spectra, revealing similar values of 3.0 and 3.4 eV for the 4:1 and 32:1 nano-SnO_2_–GO, respectively. These values are lower than the band gap of pure SnO_2_ of about 3.6 eV.^28,47^ Such decrease is attributable to the coupling between the white tin dioxide and the brownish GO sheets.

In order to investigate the powder performances, the nanopowders were deposited on Pt-IDEs via a scalable air-spraying method, obtaining highly homogeneous micrometric-thick films (Figures 2d–f). The cross-sectional FESEM images (Figure 2j–l) reveal a layer thickness of around 1.5–2.0 μm for both the SnO_2_ and the two SnO_2_–GO nanoheterojunctions (Table 1, sixth column). The estimated film porosities^29^ are above 90% for all materials (Table 1, seventh column), in line with the values expected for aerosol self-assembly processes.^48^ Insights into the optoelectronic properties and sensing mechanism of the nanoheterojunctions SnO_2_–GO were first obtained by investigating the photoresistive behavior under UV illumination. Lan et al.^49^ proposed a high-performance UV photodetector design by combining SnO_2_ semiconductors with three-dimensional graphene nanoflakes. The as-prepared nanocomposite films showed strong absorption in the wide UV region, owing to the presence of the three-dimensional (3D) network that efficiently suppresses the recombination of the photo-induced electron–hole pairs and resulted in a significant enhancement of the graphene–SnO_2_ photoresponse over that of pure SnO_2_. Notably, the responsivity of a 3D graphene–SnO_2_ photodetector was reported to be as high as 8.6 mA W^–1^ at a bias voltage of 1.0 V, which is around 8 times higher than that of pristine tin dioxide. Here, starting from this report, the current response was acquired by applying a bias potential of 1.0 V and by UV light irradiation at 312 nm with a light power density of 1.5 μW cm^–2^ (Figure 3b). The principal figures of merit for photodetectors are the magnitude of the photo-/dark-currents, responsivity, and detectivity (Table 2). Especially, the last parameter quantitatively characterizes the photodetector performances.^34^ Among the investigated samples, the 32:1 SnO_2_–GO shows the highest detectivity of 1.4 × 10^15^ Jones followed digressively by SnO_2_ and 4:1 SnO_2_–GO (Table 2, eighth column). The photocurrents, together with *I*_photo_/*I*_dark_ ratios, follow the same trend (Table 2, third and fourth columns), showing a very high *I*_photo_/*I*_dark_ value of around 2400 for the 32:1 SnO_2_–GO. Furthermore, the rise and decay times (Table 2, fifth and sixth columns) were comparable to some of the best performing SnO_2_-based UV photodetectors.^34,50^ Here, the responsivity of the 32:1 SnO_2_–GO ratio is 400 A W^–1^ and is thus very high (Table 2 and Figure 3b) with respect to the recent literature.^49^ Similarly, the 32:1 SnO_2_–GO nanoheterojunction detectivities (Table 2, eighth column) are greater than those of some of the most performing materials.^49,51,52^ This photoresponsivity trend (32:1 > SnO_2_ > 4:1) and the very high responsivity/detectivity measured with the 32:1 ratio suggest a potential mechanism for the enhancement of the UV light sensing.^53^ In line with the previous literature,^13,28,29,54^ we suggest that a p–n-type nanoheterojunction is formed between the GO, showing a p-type behavior, and the n-type SnO_2_.^31^

**Table 2:** Figures of Merit of Sn-Based Photodetectors (λ = 312 nm, Light Power Density, 1.5 μW Cm^–2^, and Applied Bias, +1.0 V)

sample	dark-current (nA)	photocurrent (μA)	*I*_Photo_/*I*_Dark_	rise time (s)	decay time (s)	responsivity (A W^–1^)	detectivity (jones)
SnO_2_	540	58	108	≈160	≈130	100	1.5 × 10^14^
4:1 SnO_2_–GO	1	0.057	52	≈130	≈110	0.100	3.4 × 10^12^
32:1 SnO_2_–GO	100	240	2380	≈120	≈100	395	1.4 × 10^15^

Upon UV light illumination, photogenerated electron–hole pairs are formed, which are rapidly separated by the SnO_2_–GO nanoscale heterojunctions, which is a disadvantage for their recombination. This results in a higher photocurrent response especially for the 32:1 ratio, where an optimal distribution of the GO in the metal oxide nanoparticles matrix may be obtained (Figure 2). The above mechanism may also be exploited to achieve high chemical sensing at RT under light illumination. Once generated, the photoelectrons (e_*h*ν_^–^) are mostly trapped on the metal oxide surface, giving rise to reactive photoinduced oxygen ions (as O_2_^–^_*h*ν_). Hence, when reducing VOC molecules are purged into the chamber, they can be oxidized by these oxygen ions releasing electrons back to the conduction band of SnO_2_ and thus increasing the film conductivity. A schematic illustration of such mechanism is reported in Figure 3c.

**Figure 4:**
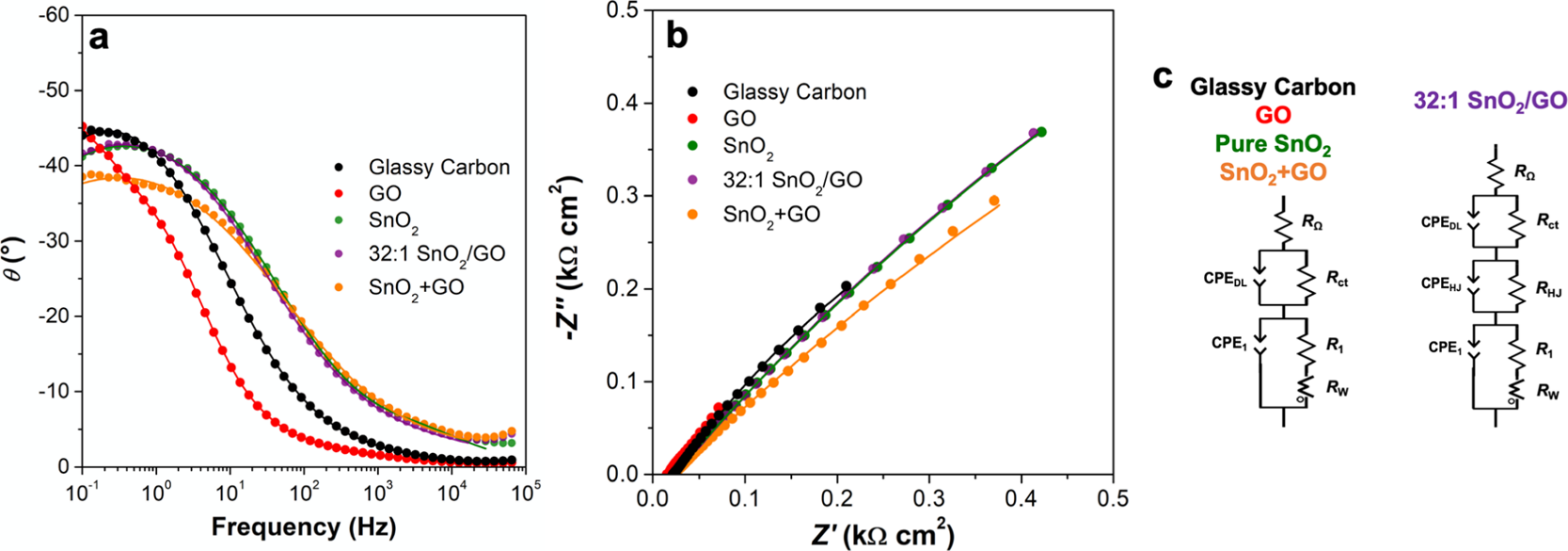
Impedance (a) Bode and (b) complex plane plots recorded for glassy carbon, GO, pure SnO_2_, 32:1 SnO_2_–GO, and mechanically mixed SnO_2_ + GO recorded in 0.1 M phosphate-buffered saline (PBS) at −0.15 V (potential at which the adopted probe, [Ru(NH_3_)_6_]Cl_3_, is oxidized). Points are the experimental values, while continuous lines are the simulated data according to the equivalent circuits, shown in (c).

Therefore, to highlight the intimate interaction between GO and MOS nanoparticles, proving the existence of optimally integrated p–n heterojunctions, EIS measurements were performed (Figure 4 and Table 3), investigating the glassy carbon/MOS/electrolyte interfaces. The computed equivalent circuits are shown in Figure 4c. Remarkably, for all the studied materials, a series resistor (*R*_Ω_, *ca*. 15–20 Ω cm^2^) was introduced to describe the electrolyte resistance and a *R*_ct_/constant phase element (CPE_DL_) parallel circuit was necessary to model the electrode/electrolyte double layer (*R*_ct_ is the charge-transfer resistance, whereas CPE_DL_ represents a nonideal double-layer capacitance). A CPE was used instead of a real capacitance because of the presence of defects that introduce inhomogeneities in the electrical material properties. Going into detail, the charge-transfer resistance (*R*_ct_) at the solid–liquid interface has similar values for the 32:1 SnO_2_–GO (0.90 kΩ cm^2^) and GO (0.03 kΩ cm^2^), and it is much smaller with respect to either the SnO_2_ (around 3.90 Ω cm^2^) or the mechanically mixed, SnO_2_ + GO (*ca*. 3.76 Ω cm^2^, Table 3) ones. Besides, the CPE_DL_ is very high for the conductive GO material (14 mF cm^–2^) and quite low for the bare SnO_2_ (*ca*. 0.2 mF cm^–2^). Interestingly, the hybrid materials exhibit an intermediate behavior, resulting in capacitance of about 4 mF cm^–2^, which is higher than that of SnO_2_ + GO (2 mF cm^–2^, Table 3). As already reported in previous works,^29,55,56^ the impedance spectroscopy technique can provide information about the actual presence of a p–n heterojunction, modeling it as a parallel combination of resistance (*R*_HJ_) and CPE_HJ_. In particular, the former is connected to the leakage and recombination paths through the p/n-type MOS interface, whereas the latter results from the nonideal capacitance due to the depletion region of the p–n junction. Remarkably, only for the 32:1 SnO_2_–GO, an additional *R*_HJ_/CPE_HJ_ circuit was added to better fit the EIS data. Finally, a third circuit (*R*_1_/CPE_1_, *i.e.*, the polarization capacitance) is present due to the interface between the powders and the glassy carbon support. CPE_1_ values similar or higher than that of the bare glassy carbon indicate the easiness of the polarization processes, as in the case of GO and the hybrid material. Furthermore, an open Warburg element (*R*_W_) was added in the fitting circuits of GO, 32:1 SnO_2_–GO, and mechanically mixed SnO_2_ + GO to take into account the probe mass-transfer process. Hence, we can infer that 32:1 SnO_2_–GO EIS behavior is quite different from the one obtained with the mechanically mixed SnO_2_ + GO compound, thus resulting in a peculiar and specific feature.

**Table 3:** EIS Fitting Parameters According to the Computed Equivalent Circuits at −0.15 V. Supporting Electrolyte: PBS 0.1 M, pH 7.4. Adopted Probe: [Ru(NH_3_)_6_]Cl_3_, 3 mM

modified-GCE	*R*_Ω_ (Ω cm^2^)	*R*_ct_ (kΩ cm^2^)	CPE_DL_ (mF cm^–2^)	*R*_HJ_ (Ω cm^2^)	CPE_HJ_ (mF cm^–2^)	*R*_1_ (kΩ cm^2^)	CPE_1_ (mF cm^–2^)	*R*_W_ (Ω cm^2^)
bare	21.9	2.95	1.4	-	-	2.0	2.0	-
GO	15.7	0.03	13.7	-	-	4.5	2.0	0.02
SnO_2_	20.2	3.90	0.2	-	-	2.4	2.1	-
32:1 SnO_2_–GO	20.5	0.90	4.0	3.6	0.03	2.5	2.2	0.05
SnO_2_ + GO	19.7	3.76	1.2	-	-	3.7	2.1	0.11

**Figure 5:**
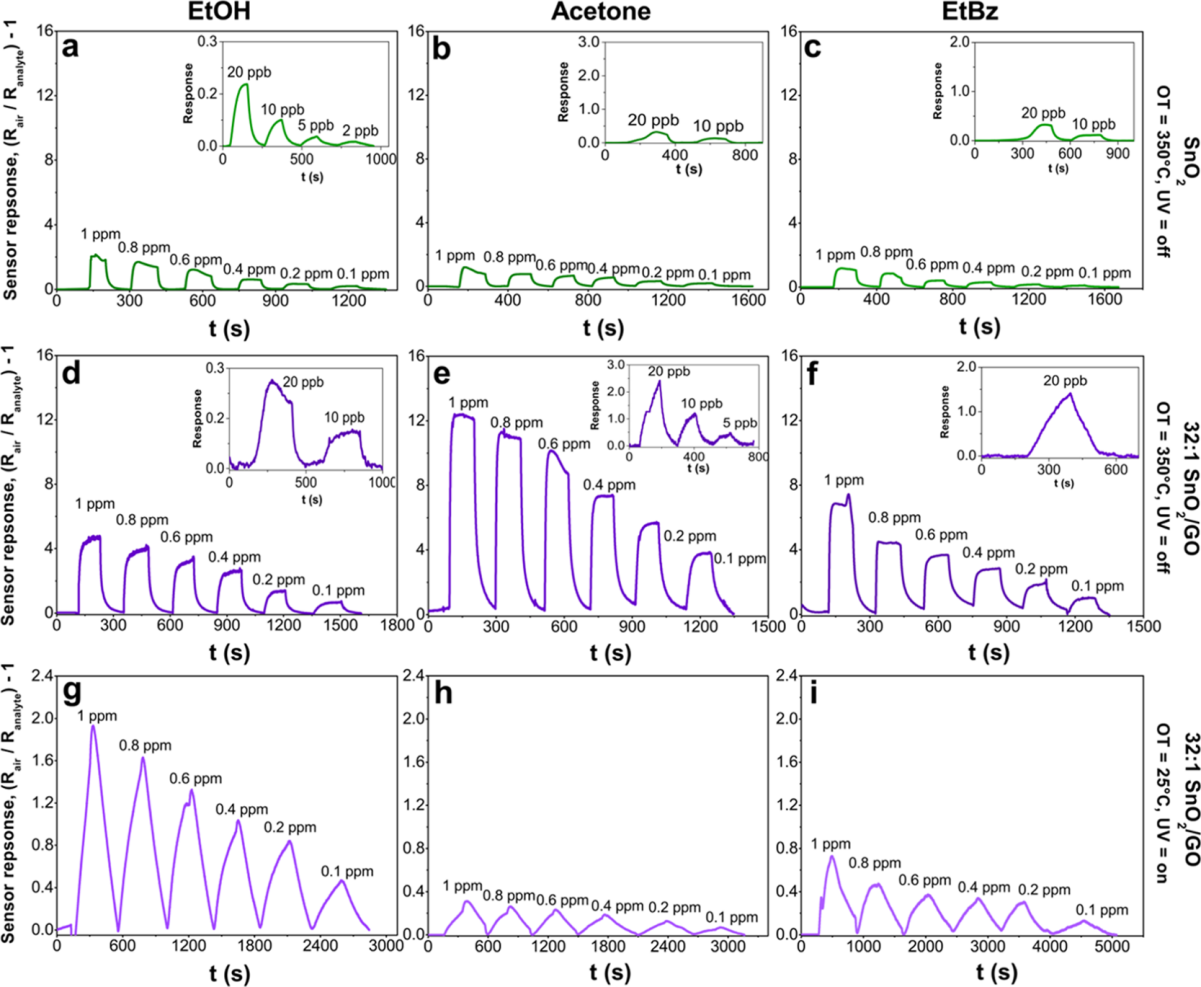
(a–c) Pure SnO_2_ and (d–f) hybrid 32:1 SnO_2_–GO sensors response when exposed to different low-ppm concentrations of ethanol, acetone, and ethylbenzene at 350 °C without UV light. (g–i) Same tests performed with hybrid 32:1 SnO_2_–GO materials at RT, UV-assisted. All the measurements were carried out in simulated air (20% O_2_–80% N_2_). OT = operating temperature.

Then, to investigate the use of optoelectronic properties of the SnO_2_–GO nanoheterojunctions for gas sensing, here, ethanol, acetone, and ethylbenzene gases were chosen as VOC model molecules. Figure 5a,d,g shows the sensor responses of the pure SnO_2_ and the optimal 32:1 SnO_2_–GO nanoheterojunction as a function of both OT and UV irradiation. Notably, at a high temperature (350 °C) without UV light, both the pure and hybrid samples can detect ethanol in air below to 2 and 10 ppb concentrations, respectively. Remarkably, for an ethanol concentration of 1 ppm, the signal intensity of 32:1 SnO_2_–GO is about 3 times higher than that of SnO_2_ (Figure 5a,d). By decreasing the temperature to 150 and 25 °C, only the nanoheterojunction was able to sense ethanol, even if light irradiation was required (Figures 5g and S3a), whereas no response was obtained for the bare oxide compound at RT with and without light irradiation. Notably, the 32:1 SnO_2_–GO had a very good signal-to-noise ratio (SNR) down to 100 ppb at RT. Furthermore, the selectivity of the materials was investigated using different VOC molecules. Acetone and ethylbenzene were used as alternative VOCs (Figures 5b,c,e,f,h,i and S3). An analogous sensing behavior was observed for these species, achieving detection at RT of 100 ppb. However, the signal intensity was quite different (Figure S4a), thus resulting in a possible selective detection of ethanol among the studied VOCs. Outstandingly, at RT, ethanol results in the highest signal response intensity of *ca*. 2 at 1 ppm, while acetone and ethylbenzene showed a lower value of about 0.3 and 0.8, respectively. This trend may follow the VOC chemical structure, that is, the presence of polar groups (such as hydroxyl groups) or steric hindrance (as the phenyl ring), thus leading to different affinities and reactivities with the oxide surface.^57−60^ It has been previously reported that alcohols are highly sensed by metal oxides rather than aldehydes or ketones and to a greater extent with respect to nonpolar/low polar analytes, such as ethylbenzene.^59−62^

Moreover, both SnO_2_ and 32:1 SnO_2_–GO readily respond and recover upon purging these three analytes with response and recovery times below 80 s at 350 °C (Figure S4b,c). Reducing the OT increases the response time by three/four times, depending on the VOC molecule.

Additionally, as clearly visible in Figure 5g–i, notwithstanding the worst operative conditions (RT and UV light), the profile of the sensing curves is satisfactory. To clarify this point, we computed the SNR for 32:1 SnO_2_–GO, as a representative example, toward the three VOCs at RT. SNRs were calculated using the following equation^63^
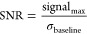
where signal_max_ is the maximum intensity of the signal and σ_baseline_ is the standard deviation in the resistance baseline before the analyte flux (calculated on at least 10 data points). Considering the limit of detection of 100 ppb (as the lowest detectable concentration at 25 °C), the SNRs values of 65, 70, and 40 toward ethanol, acetone, and ethylbenzene, respectively, were obtained.

Hence, we can conclude that our results are robust and fully in agreement with those already reported in the literature^63^ for optimal VOC sensing materials, evidencing that a very low detection limit can be reached toward the three investigated VOCs, even at RT, by using our nanoheterojunctions.

A comparative summary of the SnO_2_–GO sensing performances with literature data about SnO_2_-based chemoresistors^2,14,15,64,65^ is reported in Table 4. Interestingly, all the nanoheterojunctions, synthesized here, have superior performance than some of the best already reported. In particular, the 32:1 SnO_2_–GO exhibits significantly higher signal intensity with a very low limit of detection and high sensitivity even at RT.^15,65^

**Table 4:** Comparison of SnO_2_-Based Material Sensing Performances toward the Three Investigated VOCs

material	operating temperature (°C)	VOC	signal response, (R_air_/R_analyte_)–1^b^	LOD^a^ (ppb)	refs
hollow SnO_2_	300	EtOH	28.2 (100 ppm)^c^	5000	(18)
rGO–SnO_2_	300	EtOH	42.0 (100 ppm)^c^	5000	(15)
		acetone	11.0 (100 ppm)^c^	–	(15)
0.1 wt % GO/SnO_2_ nanocomposite	250	EtOH	22.5 (50 ppm)	1000	(17)
SnO_2_ hollow spheres	200	acetone	15.0 (50 ppm)^c^	5000	(19)
Rh-doped SnO_2_ nanofibers	200	acetone	59.6 (50 ppm)^c^	1000	(64)
3% CuO/SnO_2_	280	EtBz	7.0 (50 ppm)^c^	2000 of BTEX	(4)
					
SnO_2_	350	EtOH	2.0	2	this work
		acetone	1.8	10	this work
		EtBz	1.5	10	this work
					
32:1 SnO_2_–GO	350	EtOH	5.1	10	this work
		acetone	12.5	5	this work
		EtBz	7.2	20	this work
	RT (UV)	EtOH	2.0	100	this work
		acetone	0.4	100	this work
		EtBz	0.8	100	this work
					
4:1 SnO_2_–GO	350	EtOH	0.1	100	this work
		acetone	0.6	100	this work
		EtBz	0.4	100	this work
	RT (UV)	EtOH^d^	0.006	1000	this work
		acetone	–0.1	100	this work
		EtBz	–0.6	100	this work

a LOD, limit of detection.

b Always referred to 1 ppm, otherwise stated.

c Calculated from data reported in the reference.

d Ref (28).

**Figure 6:**
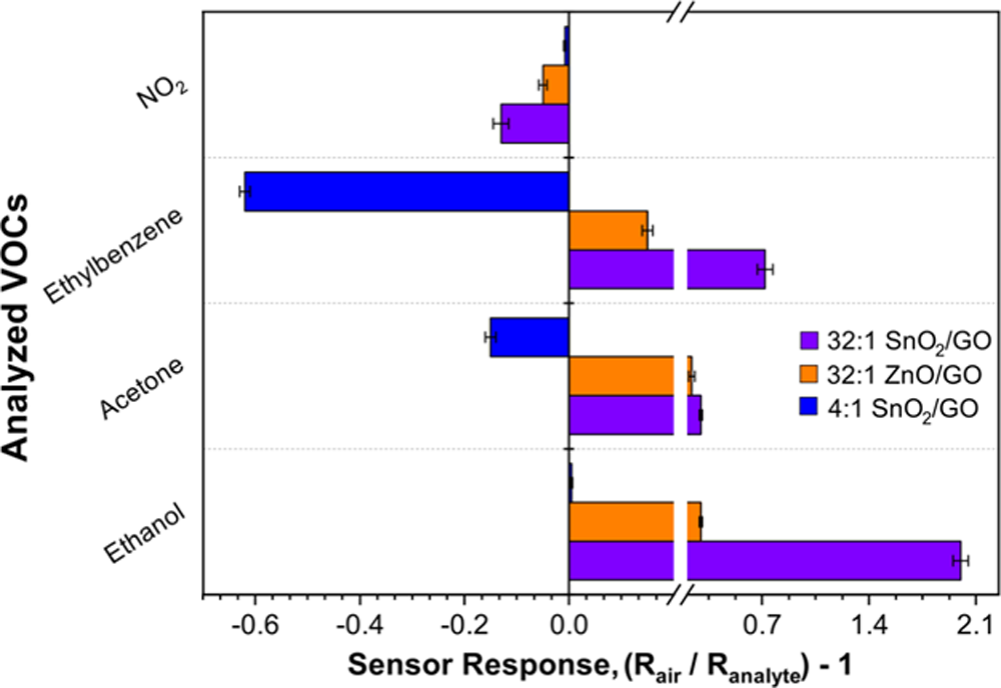
Comparison among 32:1, 4:1 SnO_2_–GO, and previously reported 32:1 ZnO–GO sensors^29^ in terms of signal response intensity to 1 ppm of NO_2_, ethylbenzene, acetone, and ethanol at 25 °C under UV irradiation.

The feasibility of tuning the chemical response of the nanoheterojunctions by engineering their composition was further obtained correlating their sensing response at a constant VOC concentration. Figure 6 shows a comparison of responses at 1 ppm relative to different nanoheterojunctions composed of 32:1 SnO_2_–GO, 32:1 ZnO–GO, previously reported,^29^ and 4:1 SnO_2_–GO. Interestingly, the 32:1 SnO_2_–GO has significantly higher ethanol selectivity than the other species, showing a response of about 4 times higher than that of acetone. These results show that at a constant relative GO amount, tin dioxide is more selective to ethanol and has significantly higher sensitivity than zinc oxide containing the nanoheterojunction. This may be attributed to the grain boundary density of the two nanoheterojunctions. The change in material resistance depends mainly from the ratio between the grain size (*d*) and the Debye length (δ).^66^ If *d* is slightly lower or equal to 2δ, the whole grains are depleted and change in the surface oxygen species concentration can affect the entire grain, resulting in higher sensitivity. Here, the particle sizes of both SnO_2_–GO (∼5–8 nm) and ZnO–GO (∼50 nm^29^) compounds are very close to twice the Debye length of tin dioxide (∼3 nm^65,67,68^) and zinc oxide (∼30 nm^69^), respectively. Therefore, an improvement of the sensing behavior is expected. However, in the case of zinc oxide, Bo et al.^34^ recently reported that the further increase of ZnO nanoparticle dimensions beyond 42 nm does not help to enhance the optoelectronic features. This is mainly ascribed to the slightly greater backscattering phenomena, causing reduced photosensing performances. Furthermore, in the case of acetone and to a greater extent for ethylbenzene, we observed a reversed change in conductance with the 4:1 SnO_2_–GO sample. This phenomenon is reported to be typical of MOS operating at low temperatures because of a greater amount of adsorbed oxygen species,^70^ leading to a more hydrophilic surface. In this sample, indeed, the incomplete GO coverage results in a greater adsorption of oxygen species and moisture with respect to the 32:1 SnO_2_–GO. In order to demonstrate the conductivity switching at low temperature, tests at high temperatures were carried out (Figure S5). We observed that the signals both for acetone and ethylbenzene switch from negative to positive values by rising up the OT above 150 °C, along with an increase in the relative intensities. Because this behavior was observed for ethylbenzene molecules only in the case of 4:1 SnO_2_–GO nanoheterojunction, it can be used as a tool to selectively sense this species at RT.

Moreover, to further investigate both the selectivity of our hybrid materials to VOC compounds and the interference of water vapor molecules, sensing tests at RT toward NO_2_ gas on the one hand and at RH on the other hand were carried out.

Concerning the former, as shown in Figure S6, all the three best performing compounds, 32:1 SnO_2_–GO, 32:1 ZnO–GO, and 4:1 SnO_2_–GO, exhibited a response to 1 ppm of NO_2_. As expected for oxidizing species, we observed a current decrease in the presence of nitrogen dioxide. Notably, the signal intensity is at least around 4 times lower than the one achieved toward VOC species, confirming the higher selectivity (Figure 6). By using the best performing 32:1 SnO_2_–GO material composition, sensing measurements toward the three VOCs were performed at RH of *ca*. 80%. Figure S7 exhibits a significant decrease (50% at most) of the sensor response to all the three target gases. However, the relative selectivity toward ethanol was preserved, indicating that with an appropriate parallel measurement of the humidity level, these materials have potential for translation into commercial devices.

As a result, tailoring of the GO content in a 3D SnO_2_ network enables to achieve high sensitivities and selectivities toward different VOCs at RT.

## Conclusions

Herein, we succeeded in engineering the optoelectronic performance of SnO_2_–GO nanoheterojunctions for the selective and sensitive measurement of VOCs at RT. The effective integration of the carbonaceous material into the metal oxide network was confirmed by XRD, Raman, XPS, and high-resolution TEM analyses. Highly porous film structures, with an average thickness of around 1.5 μm, were obtained by depositing the SnO_2_–GO nanodomains onto Pt-IDEs by a scalable air-spraying method. The enhancement of the sensing performance over that of the bare SnO_2_ is attributed to the relative fraction of p(GO)–n(SnO_2_) nanodomains, which promotes the electron–hole separation. Hence, by exploiting impedance measurements, an additional *R*_HJ_/CPE_HJ_ circuit was introduced to verify and corroborate this unique behavior. Notably, we observed that the fine control of the GO amount in the SnO_2_ nanoparticle network is a potential path to tune the selectivity to VOCs. A low GO content results in an enhanced UV light responsivity of *ca*. 400 A W^–1^, with short 120 and 100 s rise and decay times, and RT detection of below 100 ppb of ethanol, with good selectivity against other VOCs such as acetone and ethylbenzene. Conversely, a high amount of GO hinders the ethanol response at RT, enhancing an opposite change of conductivity and selectivity to ethylbenzene. We proposed that selectivity switching mechanism is mainly due to the different surface compositions of the 4:1 SnO_2_–GO nanoheterojunction. The latter has a highly more hydrophilic surface than that of 32:1 SnO_2_–GO, resulting in the adsorption of moisture and hydroxyl groups at RT, which can compete with the target VOCs for adsorption sites. Moreover, further tests were carried out to investigate both the selectivity toward other interfering gases, such as nitrogen dioxide, and the role played by water vapor molecules. We observed a significantly decreased response to NO_2_, which is at least 4 times lower than the one obtained toward VOC species. Second, a RH of about 80% led to a remarkable decrease of response intensity, even if both sensitivity and selectivity were preserved. We believe that these findings provide guidelines for the engineering of miniaturized chemoresistive sensors for selective RT detection of various VOCs. The excellent performance of the SnO_2_–GO nanoheterojunctions as UV photodetectors also provides a tunable low-cost material for the fabrication of optoelectronic devices for various applications.

## Supporting Information Available

Resistance variation of SnO_2_ and 32:1 SnO_2_–GO upon purging ethanol and GO at the same operating condition as the one used during sensing measurements; BET isotherms relative to GO, bare SnO_2_, hybrid 4:1, and 32:1 SnO_2_–GO; pore volume distribution along with the relative total pore volume; hybrid 32:1 SnO_2_–GO sensor response when exposed to different concentrations of ethanol, acetone, and ethylbenzene at 150 °C with UV light; signal response *versus* different VOC concentrations with both pure and 32:1 SnO_2_–GO, response, and recovery times as a function of OTs; acetone and ethylbenzene sensing by 4:1 SnO_2_–GO sample at 350 °C without UV light, 150 °C, and RT under UV irradiation; example of 32:1 SnO_2_–GO, 4:1 SnO_2_–GO, and 32:1 ZnO–GO responses to 1 ppm of interfering species such as NO_2_ at RT by exploiting the UV light; and example of 32:1 SnO_2_–GO responses to different concentrations of ethanol, acetone, and ethylbenzene at RT by exploiting the UV light and in humid atmosphere (RH = 80%) (PDF)
