# Supplementary material for: Engineering of SnO2–Graphene Oxide Nanoheterojunctions for Selective Room-Temperature Chemical Sensing and Optoelectronic Devices
Source: ACS Appl Mater Interfaces. 2020 Jul 22;12(35):39549–60. doi: 10.1021/acsami.0c09178 (PMC8009473; doi:10.1021/acsami.0c09178)

Supporting Information

Engineering of SnO_2 – Graphene Oxide Nano-Heterojunctions for Selective Room-Temperature Chemical Sensing and Optoelectronic Devices

Eleonora Pargoletti,[1,2] Umme H. Hossain,[3] Iolanda Di Bernardo,[4,†] Hongjun Chen,[4] Thanh Tran-Phu,[4] Gian Luca Chiarello,[1] Josh Lipton-Duffin,[5] Valentina Pifferi,[1,2] Antonio Tricoli[4*] and Giuseppe Cappelletti[1,2*]

[1] *Dipartimento di Chimica, Università degli Studi di Milano, via Golgi 19, 20133, Milano, Italy*
[2] *Consorzio Interuniversitario Nazionale per la Scienza e Tecnologia dei Materiali (INSTM), Via Giusti 9, 50121, Firenze, Italy*
[3] *Department of Electronic Materials Engineering, Research School of Physics and Engineering, The Australian National University, Canberra ACT 2601, Australia*
[4] *Nanotechnology Research Laboratory, College of Engineering and Computer Science, The Australian National University, Canberra ACT 2601, Australia*
[5] *Institute for Future Environments (IFE), Central Analytical Research Facility (CARF), Queensland University of Technology (QUT), Brisbane, Australia*
[†] *Present Address: ARC Centre of Excellence for Future Low-Energy Electronics Technologies (FLEET), School of Physics and Astronomy, Monash University, Melbourne VIC 3800, Australia*

e-mails: giuseppe.cappelletti@unimi.it; antonio.tricoli@anu.edu.au

Figure S1. Sensor response in terms of resistance values for (a) bare SnO_2 and (b) hybrid 32:1 SnO_2/GO (as representative sample) at 350 °C, without UV light and with applied bias of +1.0 V, upon purging different concentrations of ethanol molecules. Resistance values of (c) GO at the experimental conditions of the sensing measurements (RT, UV on, applied bias of +1.0 V); and (d) 32:1 SnO_2/GO upon purging ethanol molecules, at RT, with UV light and bias of +1.0 V.

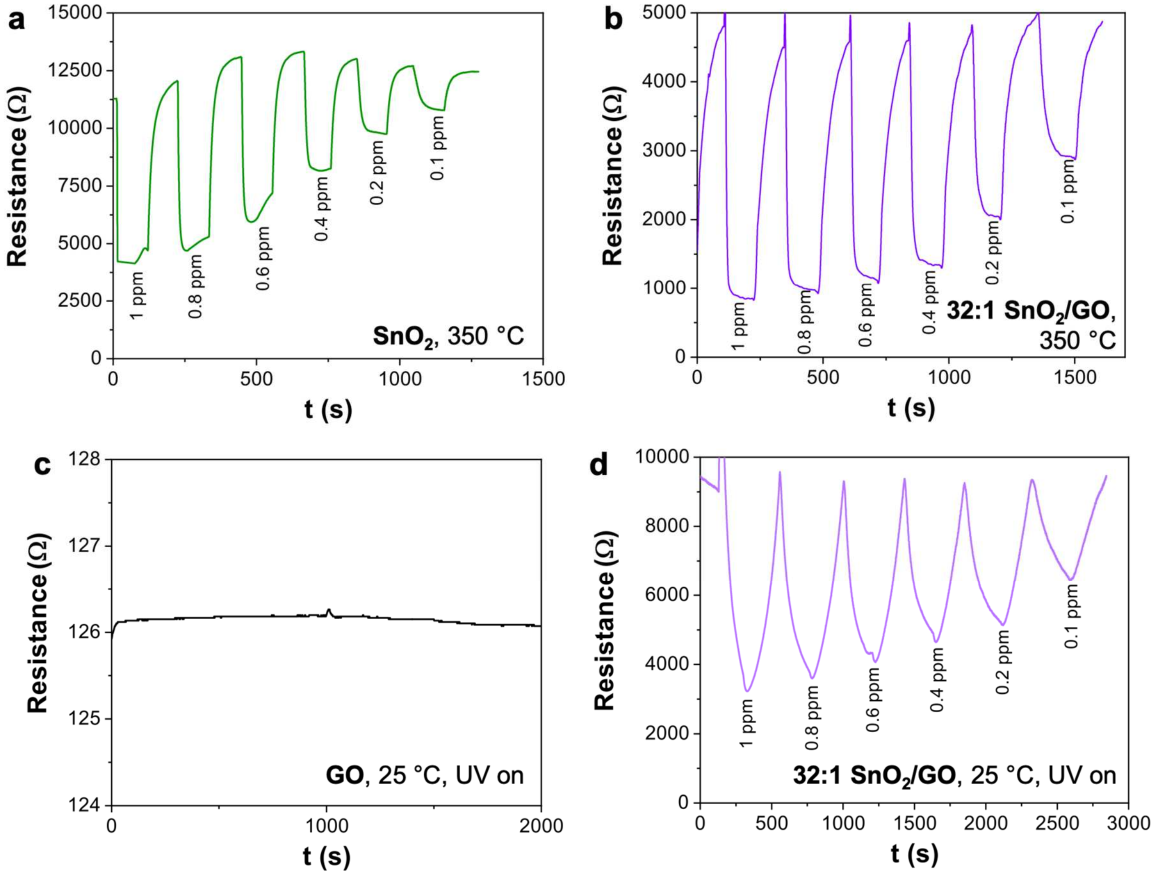

Pure SnO_2, as various metal oxides, has high resistivity which, in turn, leads to their use only at high temperatures. Since resistivity is directly proportional to the material resistance, the active sensing area and film thickness are almost the same for all the tested samples, in gas sensing field, responses can be reported as a variation of the sensors resistance upon purging a certain concentration of the target analytes. Hence, to shed light on our materials and their enhanced performances, we report in the new Figure R2 the resistance of pure SnO_2 (Fig. R2a) and 32:1 SnO_2/GO (as representative one) at both high (350 °C) and room-temperature conditions (Fig. R2b,d), alongside with the GO resistance at room temperature (Fig. R2c). For these tests, we adopted the same experimental conditions used during the sensing measurements, at 350 °C and RT, with an applied bias of +1.0 V and UV light when operating at low temperature. We observed that graphene oxide reaches a quite low resistance of about 130 Ω (Fig. R2c) even at room temperature, indicating a lower resistivity than the hybrid compounds. Nevertheless, also 32:1 SnO_2/GO sample shows a value of ca. 9 kΩ at RT, which decreases upon purging the VOC molecules, as ethanol in Figure R2d. Hence, we can conclude that our hybrid materials have peculiar electrical features thanks to the anchoring of metal oxide nanoparticles on GO sheets through its oxygen-containing functionalities. Furthermore, the free residual GO groups unbound to the MOS might have been removed by the calcination step at 400 °C, thus slightly increasing the materials conductivity.

Figure S2. (a) BET isotherms of the GO, bare SnO_2, hybrid 4:1 and 32:1 SnO_2/GO (inset: pores volume distribution obtained from the isotherms desorption curves). (b) Pores volume distribution alongside with the relative total pores volume.

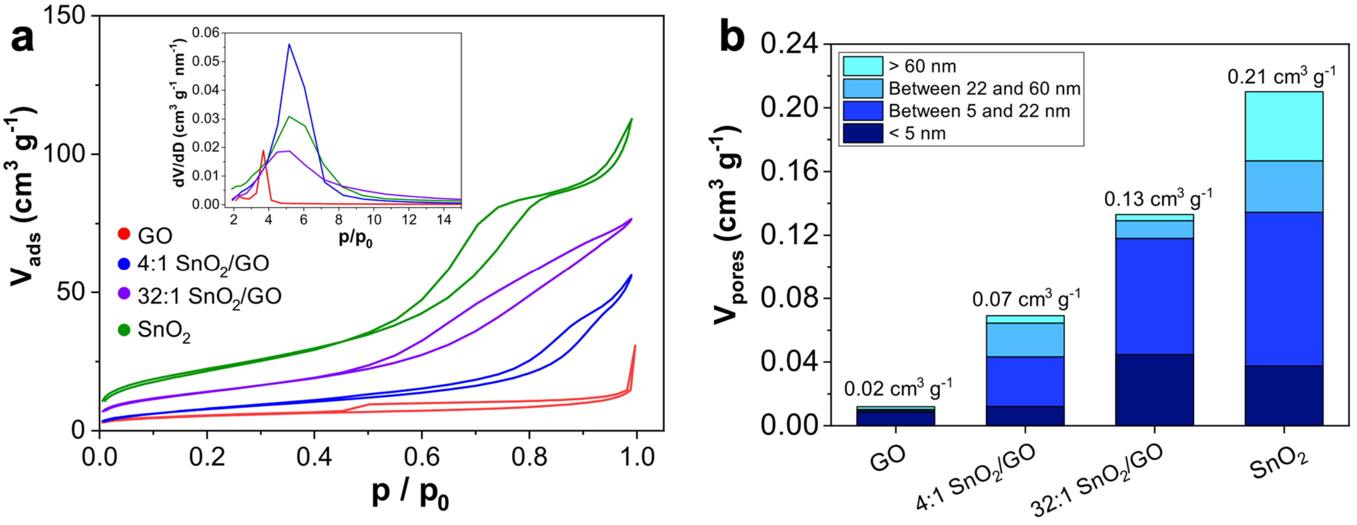

Figure S3. Hybrid 32:1 SnO_2/GO sensors response when exposed to different concentrations of (a) ethanol, (b) acetone and (c) ethylbenzene, in simulated air (20% O_2 – 80% N_2). OT = 150°C, UV-light assisted measurements.

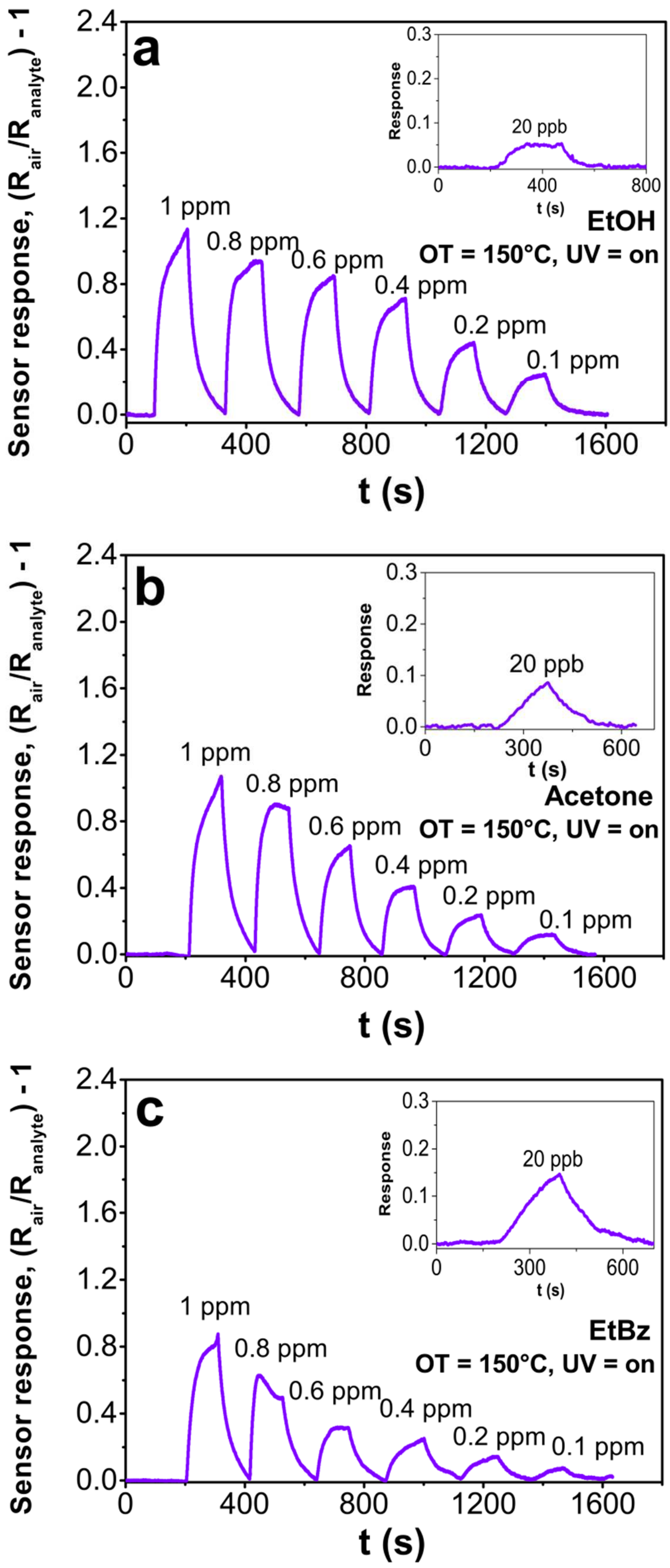

Figure S4. (a) Signal response versus different VOCs concentrations obtained with either 32:1 SnO_2/GO or pure SnO_2. (b) Response and (c) recovery times as a function of the Operating Temperature (OT).

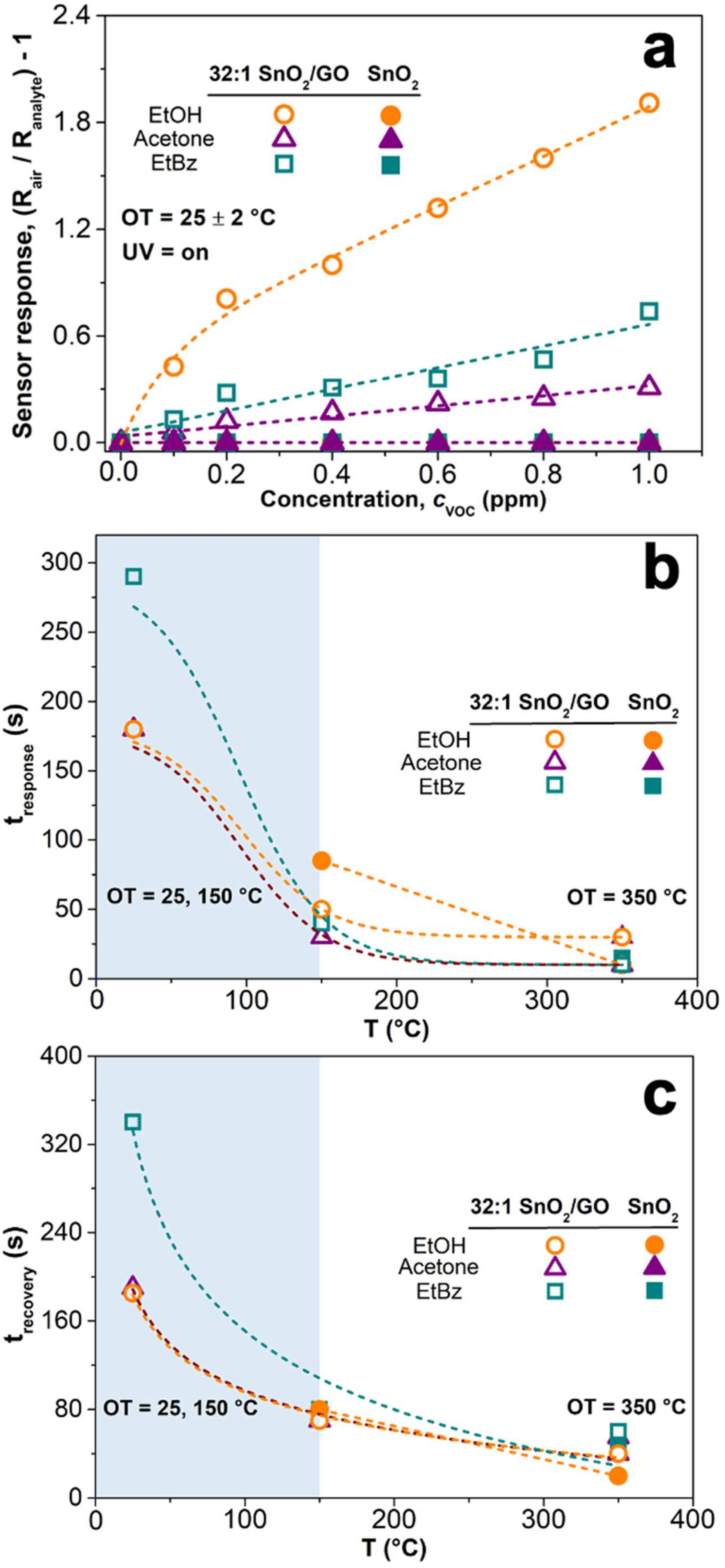

Figure S5. (a–c) Acetone and (d–f) ethylbenzene sensing by 4:1 SnO_2/GO sample at 350 °C without UV light, 150 °C and room temperature under UV irradiation.

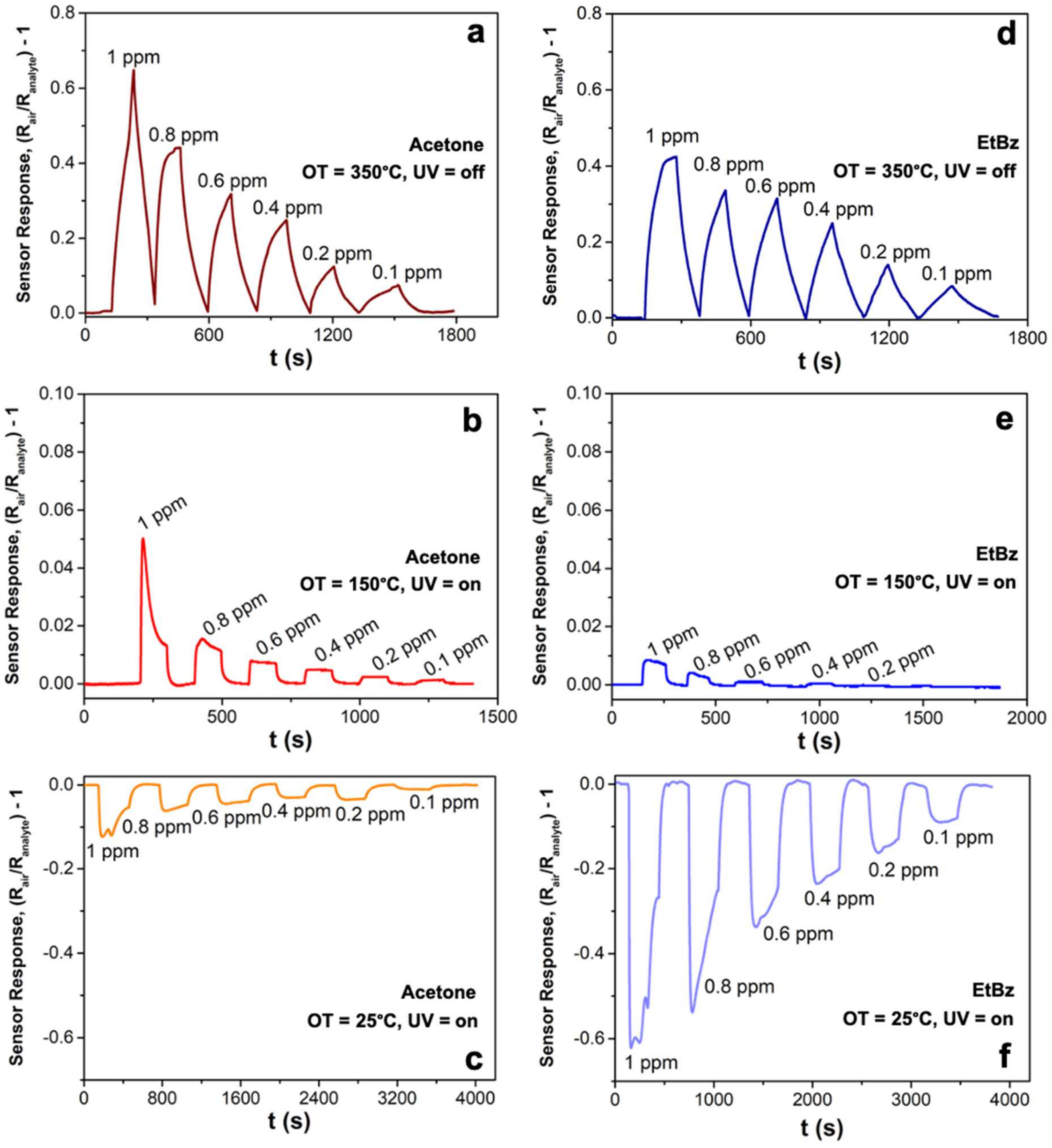

Figure S6. Example of (a) 32:1 SnO_2/GO, (b) 4:1 SnO_2/GO and (c) 32:1 ZnO/GO responses to 1 ppm of interfering species such as NO_2, at RT by exploiting the UV light. Tests were carried out in simulated air (20% O_2 – 80% N_2).

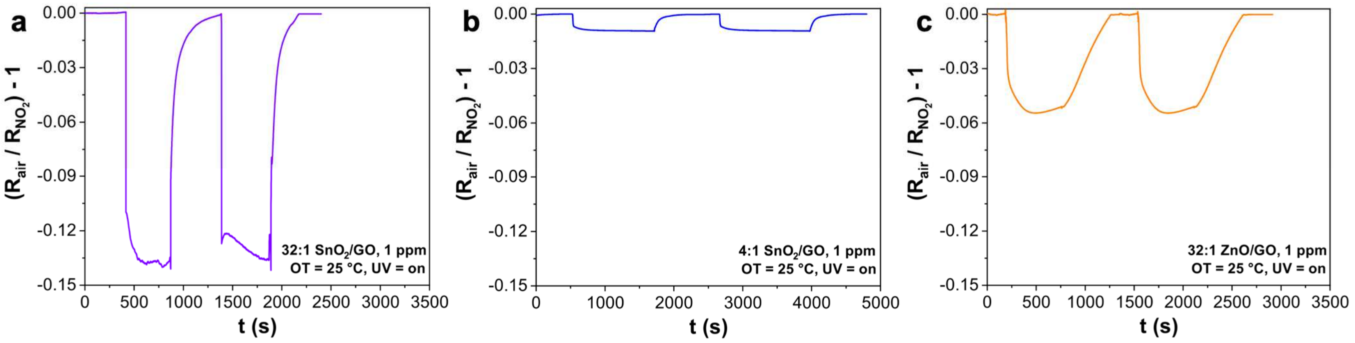

Figure S7. Example of 32:1 SnO_2/GO responses to different concentrations of (a) ethanol, (b) acetone and (c) ethylbenzene, at RT by exploiting the UV light and in humid atmosphere (RH = 80%). (d) Comparison of signals response versus different ethanol concentrations passing from RH 10% up to 80%. Tests were carried out in simulated air (20% O_2 – 80% N_2).

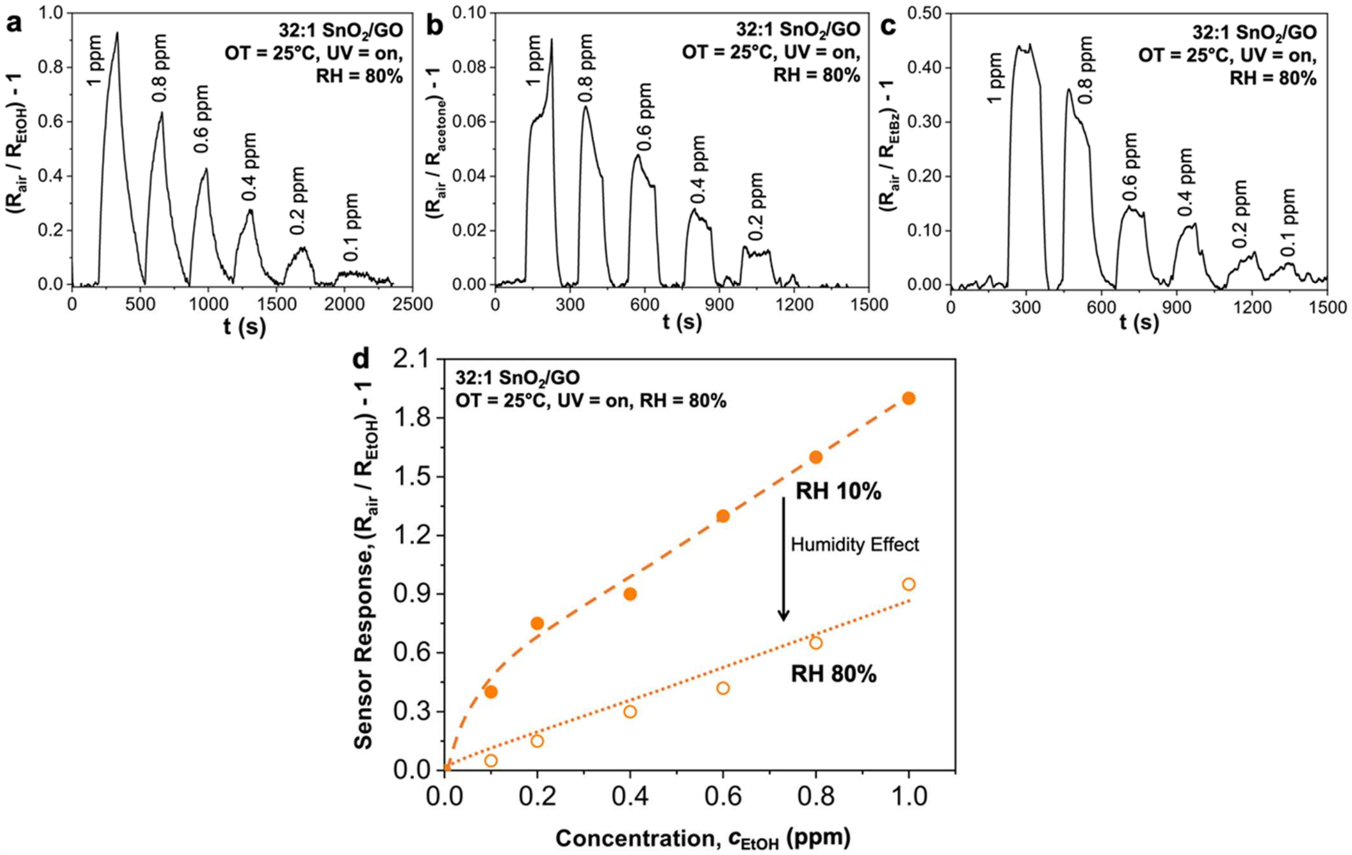

Supplement: Supplementary file 1 — am0c09178_si_001.pdf [file am0c09178_si_001.pdf]
